# Accuracy Evaluation of Thermoelastic Stress Analysis with the Use of Experimental and Numerical Methods

**DOI:** 10.3390/ma15051961

**Published:** 2022-03-07

**Authors:** Robert Misiewicz, Przemysław Moczko, Adam Bajcar

**Affiliations:** 1 Department of Machine Design and Research, Faculty of Mechanical Engineering, Wroclaw University of Science and Technology, 50-370 Wrocław, Poland; robert.misiewicz@pwr.edu.pl; 2 “Poltegor-Instytut” Institute of Opencast Mining, 51-616 Wrocław, Poland; adam.bajcar@igo.wroc.pl

**Keywords:** Thermoelastic Stress Analysis (TSA), stress field identification, numerical simulation

## Abstract

Thermoelastic Stress Analysis (TSA) is one of the very few methods allowing the determination of a continuous stress distribution on the object’s surface under variable loading conditions. Such results provide a lot of valuable information in the field of technical condition assessment and residual life prediction. In order to improve the accuracy of the TSA, the Lock-In signal processing method is implemented. This research is aimed at verifying the effectiveness of this improvement and determining the TSA stress detection threshold, as it is important information in terms of the applicability of this method in the low-stress conditions encountered in considerations of fatigue of load-carrying structures. A steel sample with a centrally located hole was subjected to cyclic loads to determine the threshold of stress detection and accuracy of TSA. As a result of the research, the relationship between the magnitude of stress excitations and the underestimation of the measured stresses was developed. Based on the conducted investigations, it was concluded that reasonable TSA results can be acquired for excitations that induce a temperature response above 10 mK (0.5 NEDT). The presented field test example proves that in industrial applications reasonable results can be acquired for thermal responses below the NEDT of the IR camera. It was concluded that it is possible to successfully implement TSA in low-stress applications (temperature response below NEDT).

## 1. Introduction

The determination of continuous stress distribution on the surface of an object under variable loading conditions is very important when it comes to condition assessment, predicting residual life, fatigue calculations, etc. There are various methods that use thermal phenomena for non-destructive materials testing and evaluation [1,2]. One of the methods that provides such data and is of interest to the authors of this paper is Thermoelastic Stress Analysis (TSA). The reason for that is that TSA is mainly used in laboratory research. Laboratory measurements provide several amenities which, in addition to stable environmental conditions, include full control of the load conditions to which the sample is subjected. To obtain high-quality results from the TSA, a measured IR signal must be of appropriate strength. In the laboratory, this is achieved by maximizing the excitation magnitude that induces stress near the elastic limit of the tested material. However, this approach cannot be used in out of laboratory industrial conditions where the stress magnitude spectrum is more complex, and in most cases, it cannot be controlled. In addition, laboratory measurements allow for a clear reference signal representing the load conditions from the universal testing machine. The reference signal is necessary for the Lock-in procedure that increases the accuracy of the TSA method. The quality of that signal affects the final quality of the analysis results. This becomes another important issue because in the vast majority of industrial applications, obtaining a reference signal from external sources such is not possible. In such a case, the “self-reference” method has to be used. The self-reference functionality is based on retrieving the reference signal needed for lock-in processing from the selected area of the recording image. The quality of this signal is not as good as obtained from the laboratory testing machines. Therefore, in such a case, a lower accuracy and detection limit should be expected.

The stress detection threshold of the Thermoelastic Stress Analysis method is important in the case of multiaxial conditions with low stress amplitude occurring in complex applications where fatigue considerations are important [1,2,3,4,5,6,7]. Nevertheless, stress detection threshold considerations are lacking. As the authors’ objective is to use TSA in real-world engineering applications, the answer to what is the stress detection threshold utilizing the “self-ref” feature, is a substantial matter that has not been investigated yet. Simple relation between the NEDT of the IR camera and the detection threshold leads to wrong conclusions in this case. Discussion on the noise equivalent temperature difference (NETD) of the TSA system can be used as a reference in the analyzed issue. However, the opinions in this matter are not consistent. According to ref. [8] the noise equivalent temperature difference (NETD) of the TSA measurement system based on Cedip Silver 480M (320 × 240, InSb 3–5 µm, NETD 20mK detector) IR camera can be reduced down to 4 mK, thanks to lock-in technique on which TSA is based [9]. According to ref. [10] thermal sensitivity is of the order of approximately 3 mK while referring to refs. [11,12] a thermal resolution of 1 mK or better can be achieved. According to cited sources, thermal noise tends to decline as a function of the increasing number of loading cycles and increasing collection integration time. These parameters can be easily set at laboratory measurements where hundreds or thousands of cycles could be acquired to provide one TSA image of exceptional quality. In the case of industrial applications, loads are not adjustable, thus the measurement system might not work as effectively as we expect. The authors noted that the literature does not mention the influence of the excitation magnitude on the quality of the results, and such relation was investigated and presented in the paper which is another research gap that the authors fill. 

The paper reveals the results of laboratory investigations aimed to evaluate the stress detection threshold for the TSA method [13,14,15,16]. Following laboratory tests, TSA results of industrial application were also provided to prove laboratory results validity.

## 2. Materials and Methods

To investigate the stress detection threshold and the influence of the excitation magnitude on TSA quality results, a set of laboratory measurements were carried out. This analysis provides a foundation for determining the possibility of using this measurement technique in research in industrial applications.

**Figure 1:**
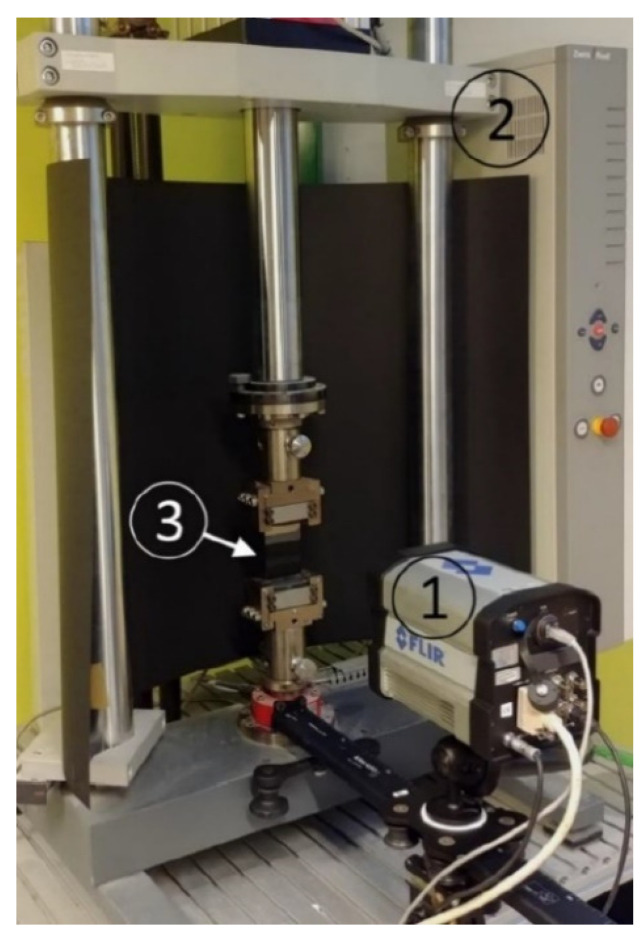
TSA experiment setup. 1—FLIR SC6000 IR camera, 2—Zwick/Rockwell Z030 tensile machine, 3—Specimen.

The measurement system is based on FLIR SC6000 InSb 3–5 µm, 20 mK NEDT infrared camera (camera manufacturer: FLIR, Wilsonville, OR, USA). The data was collected at the ambient temperature of 21 °C, at the frame rate of 90 Hz, 640 × 512 resolution, and the integration time of the detector of 2.5 ms. The experimental setup is presented in Figure 1.

**Figure 2:**
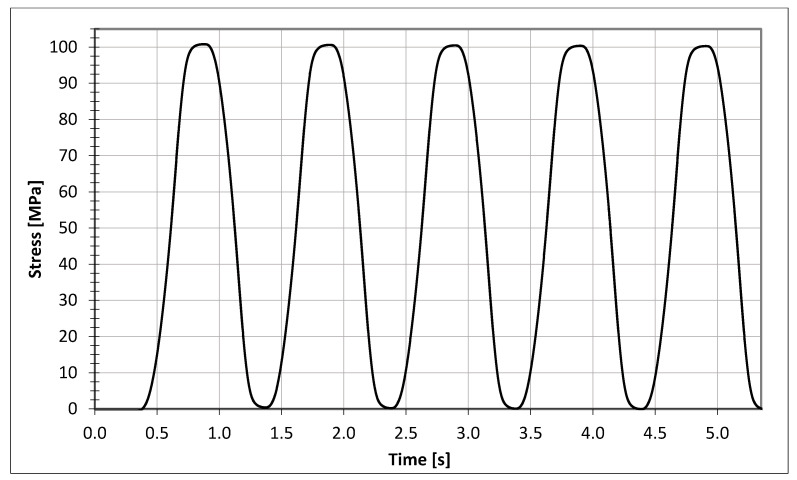
Load waveform of the 100 MPa stress range excitation.

During the measurements, the sample was subjected to a sinusoidal tensile load with a frequency of 1 Hz causing a minimum stress of 0 MPa and a maximum stress of a variable value, depending on the test. For each test, mean stress was equal to half of the stress range. An example of the load waveform is presented in Figure 2. For each TSA image, 50 load cycles were captured.

The specimen was made of S355 mild steel as it is commonly used in load-carrying structures. Additionally, S355 alloy has a stress–temperature relation close to 1 MPa–1 mK which allows to easily analyze the results in both MPa and mK. The stress–temperature relation is established by Kelvin’s equation [17,18]:

(1) $$\Delta T=\frac{\alpha \times T_{0}}{\rho \times C_{p}}\times \Delta \sigma$$

where ρ—density, C_p_—specific heat, α—coefficient of thermal expansion, T_0_—absolute temperature, Δσ—change of the sum of principal stresses, ΔT—temperature change.

The above equation allows the determination of the surface stress variations in periodically loaded material thanks to the thermoelastic effect, which describes the dependence of changes in elastic strains and temperature in solids. The foundation of this method relies on a strain-temperature relation. These changes are explained as resulting from a change in the volume due to loading of a solids within elastic stress range. As a result of tension, the volume of the object increases, which causes a decrease in its temperature, in the case of compression, the volume decreases, and temperature increases. The temperature of most solid bodies, such as metals, subjected to tension or compression within their yield strength experience a decrease or increase in temperature respectively.

Material properties used in the Equation (1) and the calculation results are presented in Table 1.

**Figure 3:**
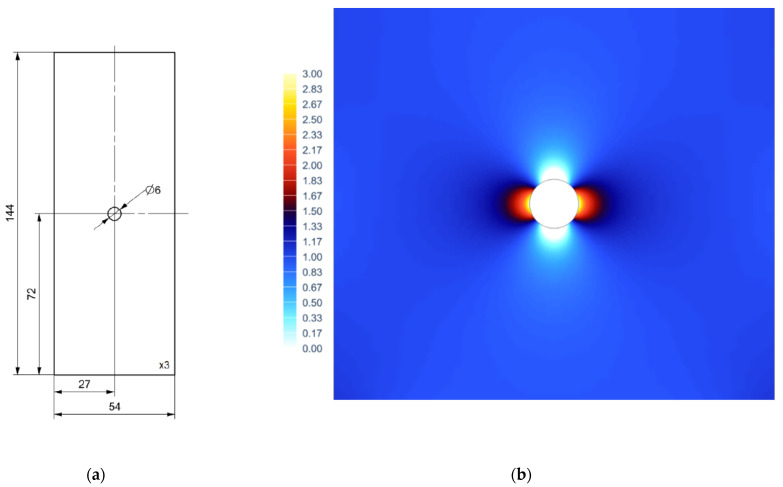
(**a**) Specimen dimensions [mm]; (**b**) FEA stress notch multiplication factor.

Specimen dimensions are presented in Figure 3a. As the TSA is readily used to determine the stress distribution in areas of stress concentration, a specimen with a notch in the form of a centrally located hole was used. The specimen itself represents Kirsch’s solution, which assumes that at the bottom of the notch stresses triples the stresses of the nominal cross-section (without the hole). Stress distribution around the hole as a notch multiplication factor was identified with the use of finite element simulation FEA (Figure 3b).

## 3. Results

**Figure 4:**
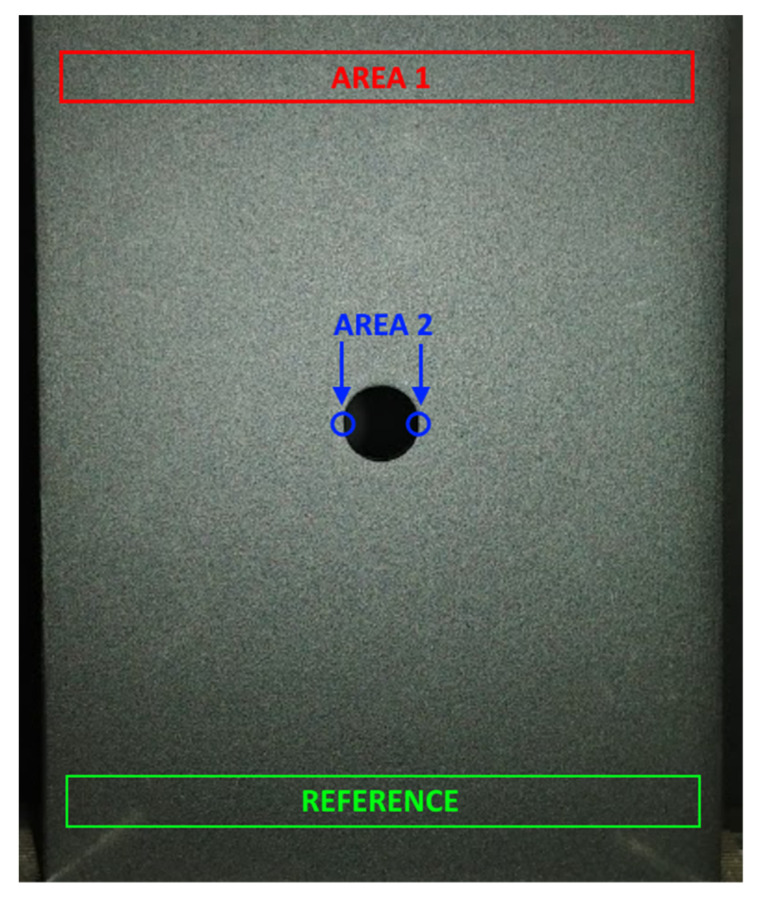
Specimen—locations of areas of interest.

To determine the detection threshold of the thermoelastic stress analysis, a series of measurements for various stress ranges were conducted. Measurements ranged from 1 to 100 MPa stress range in the nominal cross-section of the specimen. The stress ranges that the specimen was subjected to were calculated based on the force reading of the testing machine and a nominal cross-section area. Figure 4 presents the specimen and data collection areas for further analysis. For specimen loading the Zwick Z030 testing machine was used (machine manufacturer: Zwick Roell, Ulm, Germany). The reference signal for the lock-in data processing was acquired at the “reference” area, at the bottom of the specimen, using the “self-reference” approach [19,20]. This type of processing assumes that the signal obtained in the “reference” area represents load conditions and that temperature changes synchronous with this signal are searched for by the TSA system. This type of processing was chosen to represent unfavorable industrial conditions at which high-quality reference signal cannot be obtained from other measurement instruments, for instance from laboratory tensile machine. “Self-reference” configuration provides results as a multiplication of reference value which is an average value of signal acquired at the “reference” area. The data for detailed analysis were collected from two areas of interest (Figure 4):

- “area 1” which is on the opposite side of the “reference” area.
- “area 2” located at the bottom of the stress concentration notch.

**Figure 5:**
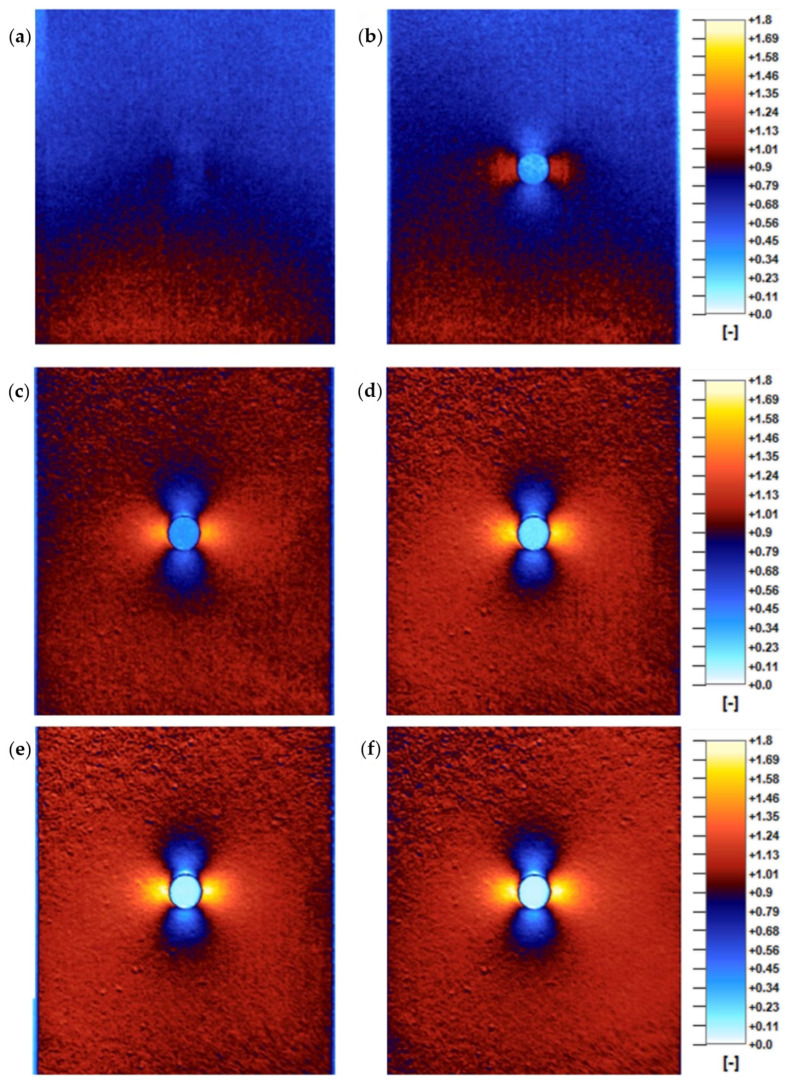
TSA results of (**a**) 2 MPa, (**b**) 4 MPa, (**c**) 10 MPa, (**d**) 20 MPa, (**e**) 40 MPa, (**f**) 100 MPa excitation, the same scale factor for all images.

The image results of the analysis are presented in Figure 5. The TSA images are an averaged thermal response of 50 loading cycles.

Selected TSA results for 2, 4, 10, 20, 40, and 100 MPa stress range excitation in the nominal cross-section of the specimen (at the “reference” area) are shown in Figure 5. The results indicate that there is a relation between excitation magnitude and TSA results quality.

For the excitations in the 1–3 MPa stress range, there was no valid data acquired. The measurement system had difficulty in distinguishing the specimen from its background what is presented in Figure 5a.

Excitations of 4 MPa are sufficient to partially recognize the specimen and the stress concentration regions, but in general, the quality of the analysis is very low. It is worth noting that there is an anomaly present in the stress pattern. It consists of the asymmetry between the “reference” area and “area 1” (Figure 4), which is an improper stress distribution for a specimen of such symmetrical geometry and implemented loads. This anomaly decreases as the excitation increases. In the range between 4–10 MPa (Figure 5b,c). there is a significant results quality improvement.

In the range between 10–20 MPa (Figure 5c,d), the anomaly is still present, but its intensity is not that significant. In this range, a positive correlation between the quality of the results and the excitation magnitude is still present, however, the improvement is not as significant as in the 4–10 MPa range. The overall quality of the results in the 10–20 MPa region is satisfactory.

For the excitation range of 20–100 MPa (Figure 5d–f) no significant quality improvements were discovered. More detailed analysis of the obtained results is presented in the next chapter.

## 4. Discussion

**Figure 6:**
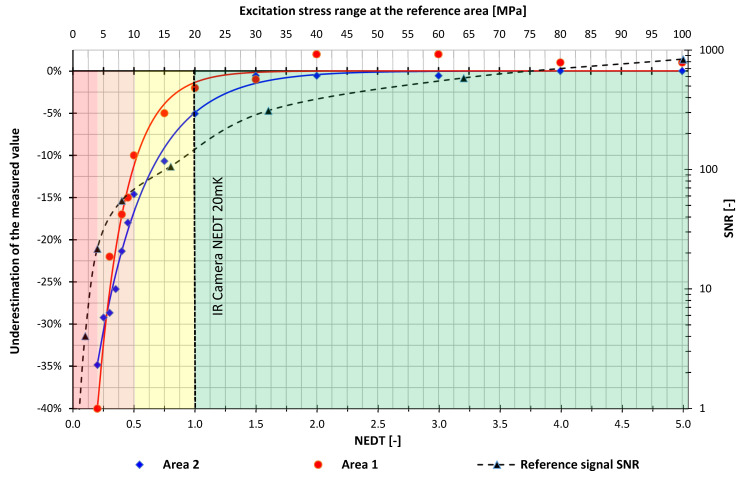
Underestimation of measured value–stress range chart.

To quantify the measured results the chart of an underestimation of the measured value in regards to the excitation magnitude is presented in Figure 6. The chart also presents the Signal to Noise Ratio (SNR) of the self-reference signal acquired at the “Reference” area. The red and blue lines present the “area 1” and “area 2” results respectively. The presented graph may explain why the opinions about the accuracy and detection threshold of the TSA technique, presented in the introduction, are not consistent. The graph shows that there is an asymptotic correlation between the measured values (area 1 and area 2 for this analysis) and the excitation magnitude. Therefore, there is no unequivocal answer on this matter.

The authors introduced four characteristic zones that can be identified in the chart (Figure 6):

- The red zone (<4 MPa)—at which it is not possible to perform TSA.
- The orange zone (4–10 MPa)—at which data acquisition is possible but the obtained results are of low accuracy. The main reason for this is the presence of significant stress pattern anomaly (“reference” vs. “area 1”) and high underestimation of measured value ranging from ~ −40 to −15%. In this zone, there is a strong positive correlation between accuracy (expressed as underestimation of the measured value) and excitation magnitude.
- The yellow zone (10–20 MPa)—at this zone stress pattern normalize and accuracy–excitation relation flattens. In this range, fairly accurate results are to expect. Some accuracy issues may still occur but the underestimation is not greater than ~ −15%.
- The green zone (>20 MPa)—this zone shows no accuracy and quality issues. Increasing the excitation magnitude seems to have no significant effect.

Stress pattern anomaly is a concerning issue as it might provide misleading results and the conclusions from the analysis. As the possible causes of anomalies related to the testing setup were investigated and foreclosed, the authors deem that the appearance of this phenomenon is associated with insufficient strength of the measured signal in low-stress ranges (<20 MPa/20 mK). In the authors’ opinion, the measurement system has a problem with distinguishing changes in temperature across the specimen surface, which are in phase with the reference signal, and a noise causing the anomaly. Figure 6 also presents the Signal to Noise Ratio (SNR) of the reference signal, the SNR correlation to “area 1” and “area 2” curves is an argument for a presented explanation. To a certain level, the quality of the analysis increases with the Signal to Noise Ratio increase. The results indicate that for SNR < 20 it is not possible to acquire valid data (the red zone) and for SNR being in the range of ~ 20–150 (the orange and yellow zone) there is a positive correlation with analysis quality. Higher SNR seems to have no significant effect on the quality of the result.

It is also interesting that “Area 2” reaches the correct value before “Area 1”. The difference may be related to the Instantaneous Field of View (IFOV), optical aberration, and the thermal signal gradient. The signal from “Area 2” is at the same time homogeneous (no gradient) and captured by many IR camera pixels. Both factors tend to stable the readings. The situation in the notch (Area 1) is different as the measurement area is much smaller (readings from fewer pixels are less accurate) and there is a signal gradient between the boundary of background and specimen notch. Optical aberration may result in the background radiation partially incident on pixels measuring notch signal resulting in lowering the reading.

There is a consensus that the “Lock-in” signal processing allows to increase the sensitivity of the TSA method below the NEDT of the IR camera and this is also confirmed by the presented results. However, in the case of the measurement configuration used, it is observed that for excitations causing the temperature response of the specimen below 20 mK (~20 MPa for mild steel) the accuracy is lower than for the excitations above 20 mK. Moreover, for excitations causing a thermal response below 20 mK, along with a decrease in the thermal response, a decrease in measurement accuracy is observed. The 20 mK (~20MPa) threshold undoubtedly seems to be correlated with the NEDT of the used camera which is rated at 20 mK, please notice the bottom axis of Figure 6 is scaled in multiples of NEDT. This leads to the expected conclusion that the best accuracy and quality of the results using “self-ref” are obtained with responses above NEDT of the IR camera. However, the range of 10–20 mK (0.5–1.0 NEDT) still seems to provide the results of acceptable quality and accuracy, but an underestimation of up to 15% is to be expected. This is important information for researchers who can estimate expected errors using the results presented in this paper.

## 5. Field Test

The examination of the stress detection threshold of the Thermoelastic Stress Analysis is a part of the research related to condition assessment and residual life prediction of heavy industrial machinery structures. A similar research approach was presented in ref. [21] where the authors conducted a set of laboratory tests to assess pipeline stresses using TSA application. Based on the laboratory results presented in the previous section it was possible to determine where the TSA method could be implemented and thus fulfill the aims of the research.

The results of laboratory tests indicate that in the case of S355 steel for stress changes below 10 MPa TSA provides rather unreliable results and using it below this threshold may result in erroneous analysis (stress pattern anomaly and significant underestimation of the measured value). These issues could potentially be solved by increasing the number of load cycles and by using external reference signal for the TSA Lock-in procedure, but as these parameters are out of control in industrial applications, the considerations of this matter were beyond the scope of this research. Considering the above facts, the place of significant stress changes has to be investigated.

**Figure 7:**
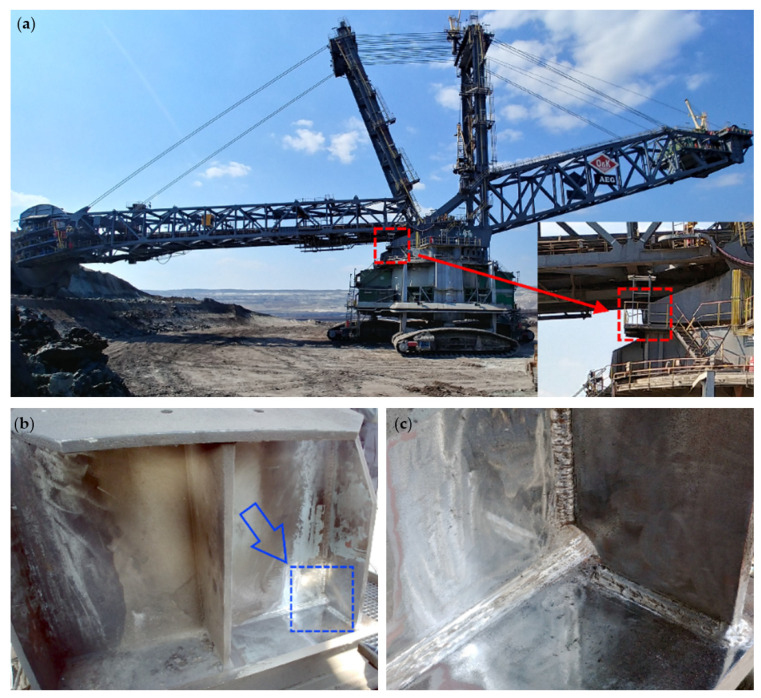
Structure under investigation: (**a**) general view; (**b**) the joint; (**c**) research area.

The tested structure and the joint chosen for investigations are presented in Figure 7. The measurements were made at the connection joint of the upper superstructure with the lower substructure of the bucket wheel excavator, as it is a place of significant load transfer and potential high strain changes during operation.

The loading spectrum of such a machine is related to the excavation process and because the material being excavated is not homogenous, the excitation spectrum is mainly random what makes the analysis difficult. Even though some of its parts tend to be harmonious and these are the parts where the TSA can be successfully implemented. Power Spectral Density (PSD) analysis performed on harmonious parts of the measured signal indicated 1 Hz as a dominant loading frequency.

**Figure 8:**
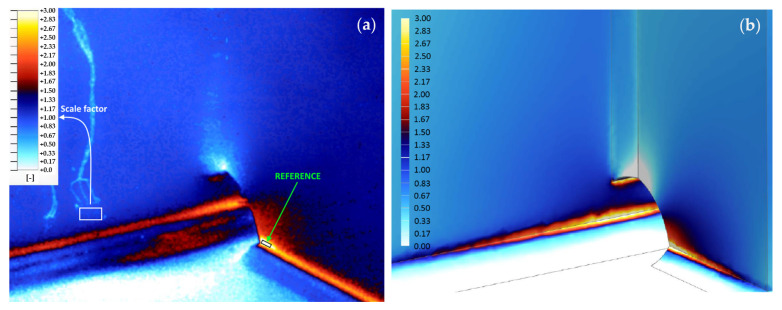
Comparison of the stress multiplication factor results: (**a**) obtained from the TSA; (**b**) obtained with the use of FEA.

The TSA measurements were processed based on the “self-referece” feature. The average stress change at the reference area (Figure 8a black rectangle), which was used to obtain the reference signal was 17.5 MPa. This value was determined based on the average stress range measured at the strain gauge point location (Figure 8a white rectangle) and then multiplied by the value of scale factor (obtained from TSA) at the “reference” area placed in the notch (Figure 8a, black rectangle). The use of a strain gauge as a calibration tool improves the accuracy of obtained TSA results. The authors of this paper recommend such an approach in the complex loading conditions of industrial machinery. The structure is made out of S355 mild steel so the temperature response at this point is close to 17.5 mK. As the laboratory measurements showed that the SNR of the reference signal affects the quality of the result, the notch was chosen as a reference to maximize the SNR and improve the quality of the result.

To verify experimental results, a finite element analysis of the investigated structure was carried out. The FEA model consisted of bucket wheel boom, masts, counterweight boom, and the ring girder located below them. As the result of the PSD analysis revealed the 1 Hz being a dominant loading frequency, the modal frequency response of the structure subjected to load applied at the end of bucket wheel boom (excavation force location) with the frequency of 1 Hz was calculated and results as a stress distribution of the tested joint obtained (Figure 8b).

Both the TSA and FEA obtained stress multiplication factor distributions are very similar in TSA and FEA (Figure 8) what indicates that the acquired results are valid. There are locally restricted differences between both results within welds surface, which are related to “ideal” shape of welds considered in the FEA model, which is typical modelling simplification approach resulting from uneven shape of real weld. The presented example proves that in industrial applications reasonable results can be acquired for thermal responses below the NEDT of the IR camera. A detailed description of the field tests will be presented in a separate paper.

## 6. Conclusions

The article presents the results of the stress detection threshold investigation using the TSA method with the use of the “self-reference” functionality, which can be used in industrial applications. Finite element analyses (FEA) was used as verification tool in the investigations. The presented research complements the current state of knowledge in the field of the TSA investigations. The test results are particularly important from the perspective of using this method in low-stress change applications. Such applications include technical condition assessment, residual life prediction, and fatigue considerations of technical objects operating at regular conditions, which significantly differ from laboratory tests in which the loads are defined and fully controlled.

As a result of the research, a correlation between the magnitude of the excitations, SNR of the reference signal, and the quality of the results were determined. It was also found that after exceeding the NEDT value, further increase of the excitation size did not have a significant impact on the quality of the results. Moreover, a very interesting phenomenon was detected consisting of the formation of anomalies in the distribution of stresses in the case of low-stress changes. The authors have not met a description of this phenomenon in the literature, which allows them to conclude that it is a new and unique finding.

The presented research confirms that Thermoelastic Stress Analysis (TSA) can be used in temperature responses below the NEDT of the utilized camera. However, for the “self-referece” processing configuration, it was discovered that for responses below 10 mK (0.5 NEDT) acquired results are misleading, so it is recommended not to use the TSA in such applications. In the presented case of the measurement configuration used, it is observed that for excitations causing the temperature response of the specimen below 20 mK (~20 MPa for mild steel) the accuracy is lower than for the excitations above 20 mK. Moreover, for excitations causing a thermal response below 20 mK, along with a decrease in the thermal response, a decrease in measurement accuracy is observed. The 20 mK (~20 MPa) threshold undoubtedly seems to be correlated with the NEDT of the used camera which is rated at 20 mK. This leads to the expected conclusion that the best accuracy and quality of the results when using “self-referece” is obtained for the responses above NEDT of the IR camera. However, the range of 10–20 mK (0.5–1.0 NEDT) still seems to provide the results of acceptable quality and accuracy, but an underestimation of up to 15% is to be expected.

The general conclusion is that the detection threshold is related to the accuracy of the results expected by the researcher. Reasonable results can be acquired for excitations that induce a temperature response above 10 mK but NEDT seems to be of utmost importance as the most accurate results are acquired above the NEDT threshold.

Example of the TSA technique use in the field test is presented in chapter 5. The TSA measurements on the structural joint of the excavator were carried out to investigate possibility of the use of this technique in out of laboratory conditions. Considering conclusions from the laboratory investigations it was decided to use of a strain gauge as a calibration that improves the accuracy of obtained TSA results. Based on the obtained results such approach is recommend in the complex loading conditions of industrial machinery. As the laboratory measurements revealed that the SNR of the reference signal influences the quality of the result the notch was selected as the reference to maximize the SNR and improve the quality of the result.

To verify experimental results, a finite element analysis of the investigated structure was carried out. The FEA model of the tested joint was used for verification of experimental data. Both the TSA and FEA obtained stress distribution and values are very similar what indicates that the acquired TSA results are valid. The presented example proves that in industrial applications reasonable results can be acquired for thermal responses below the NEDT of the IR camera.

The scope of application of the TSA method can be very wide. This method is one of the very few that allows determination of a continuous stress distribution on the surface of an object. Such results provide a lot of valuable information in the field of technical condition assessment, residual life prediction, and fatigue phenomenon investigations. This method requires further research to identify and extend possible application ranges. The presented studies are part of such activities.

## Figures and Tables

**Table 1:** Material properties and calculation results.

Material	ρ	C_p_	α	T_0_	Δσ	ΔT
	[kg/m3]	[J/kg × C]	[10–6/C]	[K]	[MPa]	[mK]
Steel S355	7800	460	12	294	1	0.98

## Data Availability

The data presented in this study are available on request from the corresponding author.
